# Chronic disease research in Europe and the need for integrated population cohorts

**DOI:** 10.1007/s10654-017-0315-2

**Published:** 2017-10-06

**Authors:** Paul Brennan, Markus Perola, Gert-Jan van Ommen, Elio Riboli, On behalf of the European Cohort Consortium

**Affiliations:** 1 Genetics Section, International Agency for Research on Cancer (IARC), 150 Cours Albert Thomas, 69372 Lyon Cedex 08, France; 2 National Institute for Health and Welfare, Helsinki, Finland; 3 Department of Human Genetics, Leiden University Medical Center (LUMC), Leiden, The Netherlands; 4 Biobanking and Biomolecular Research Resources Infrastructure (BBMRI-ERIC), Graz, Austria; 5 Imperial College London, London, UK

## The burden of chronic disease in Europe

**Fig. 1:**
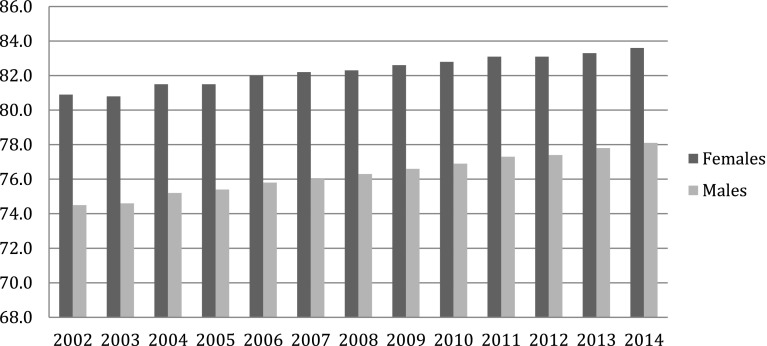
Life expectancy at birth, EU-28, 2002–2014. Eurostat Statistics Explained [2]

The burden of chronic disease in Europe is characterized by several positive trends, but also some major new challenges. On one hand there has been an important and consistent reduction in mortality, particularly in young and middle age that has led to a substantial increase in life expectancy in all 28 countries within the EU (EU28) in the last 40 years. Even within the relatively recent period of 2003–2013, there was an increase in life expectancy of 3.2 years for men and 2.5 years for women overall in the EU (Fig. 1) [1]. While these trends represent a major success in public health, they hide less positive developments, including major health disparities. Across Europe major differences in life expectancy exist, of over 10 years for men and over 7 years for women (Table 1) [1]. These differences are most extreme between southern Mediterranean Europe and the countries of central Europe and the Baltic region.

**Table 1:** Life expectancy at birth in European Union in 2014

Country	Total	Males	Females
Spain	83.3	80.4	86.2
Italy	83.2	80.7	85.6
Cyprus	82.8	80.9	84.7
France	82.8	79.5	86.0
Luxembourg	82.3	79.4	85.2
Sweden	82.3	80.4	84.2
Malta	82.1	79.8	84.2
Netherlands	81.8	80.0	83.5
Austria	81.7	79.2	84.0
Greece	81.5	78.9	84.1
Belgium	81.4	78.8	83.9
Ireland	81.4	79.3	83.5
United Kingdom	81.4	79.5	83.2
Finland	81.3	78.4	84.1
Portugal	81.3	78.0	84.4
Germany	81.2	78.7	83.6
Slovenia	81.2	78.2	84.1
**European Union**	**80.9**	**78.1**	**83.6**
Denmark	80.7	78.7	82.8
Czech Republic	78.9	75.8	82.0
Croatia	77.9	74.7	81.0
Poland	77.8	73.7	81.7
Estonia	77.4	72.4	81.9
Slovakia	77.0	73.3	80.5
Hungary	76.0	72.3	79.4
Romania	75.0	71.4	78.7
Lithuania	74.7	69.2	80.1
Bulgaria	74.5	71.1	78.0
Latvia	74.5	69.1	79.4

Bold indicates the numbers for the European Union as a whole and thus shows countries with life expectancy higher or lower than EU

Eurostat Statistics Explained [1]

Not all of this increase in life expectancy is ‘healthy life expectancy’. For example, on average in Europe, a man and a woman at age 65 will have a life expectancy of approximately 18 years and 21 years respectively. However, only 9 of these will be years lived in good health [2]. The remaining are characterized by age-related morbidity due to one or more chronic diseases (multimorbidity), resulting in an important reduction in quality of life and increasing cost to health care budgets.

Demographic changes in the age structure of the European population are also going to have an important effect on absolute numbers of disease events even assuming no major changes in age-specific incidence rates. For example, the absolute number of cancer cases in the 28 EU countries is projected to increase due to demographic effects from 2.75 million in 2015 to over 3.1 million cases per year by 2025 [3]. The number of people who are living with cancer has also been increasing. It is estimated that there are currently 16–17 million European citizens who either are being treated for cancer or are in post-treatment long term remission, and this number will also increase substantially over the next 10–20 years. Regarding specific causes of death, 85% of deaths in the EU are due to chronic diseases including cancer, cardiovascular disease, chronic respiratory disease, diabetes, and mental illness. Cancer from all causes is the predominant cause of death before the age of 65, whereas cardiovascular disease is the predominant cause of death after age 65.

## The financial burden associated with chronic disease in Europe

The financial costs associated with treating chronic diseases are extremely high, and given that the average age of European populations is increasing, chronic diseases will continue to place an important pressure on national budgets. The economic burden of cancer care on its own was estimated at 126 billion euros in 2009, approximately 40% of which was health care costs, 40% was due to productivity losses and lost working days, and 20% due to costs related to informal care [4]. Healthcare costs in the European Union currently make up between 7% and 11% of overall GDP expenditure (Table 2) [2]. Chronic diseases also have important societal costs as they depress wages, workforce participation and labour productivity, as well as increase early retirement, high job turnover and disability. Treatment costs for some chronic diseases are also rapidly increasing, especially for treatment of late stage cancers. The demand for expensive health care interventions is likely to increase substantially over the medium term in Europe at a time of limited economic growth and stagnant national health budgets. These trends represent genuine and important concerns for all national governments in Europe, and place increasing pressure on the ability to deliver sustainable health services. The co-occurrence of multiple chronic diseases in an individual, typically referred to as multimorbidity, is also becoming increasingly common, as it is more prevalent at older ages. Over 50 million people in Europe have more than one chronic disease, due to either random co-occurrence, possible shared underlying risk profile, or synergies in disease development [5]. The costs of treating and caring for patients with multiple conditions tend to increase dramatically with the number and combination of comorbidities, although the pattern varies for certain specific diseases [2, 6]. A greater understanding of the underlying causes of multimorbidity is essential in order to curb the increasing prevalence of this condition.

**Table 2:** Health care expenditures in fractions of gross domestic product (GDP)

More than 10/%		8–10%		7–8%		Less than 7%	
Country	GDP (%)	Country	GDP (%)	Country	GDP (%)	Country	GDP (%)
Netherlands	11.8	Portugal	9.7	Hungary	7.7	Luxembourg	6.8
France	11.2	Spain	9.2	Bulgaria	7.7	Lithuania	6.4
Germany	10.9	Greece	9.2	Slovakia	7.6	Poland	6.3
Belgium	10.9	Sweden	9.1	Czech Republic	7.4	Latvia	6.0
Denmark	10.6	Finland	8.7	Cyprus	7.3	Estonia	5.8
Austria	10.4	Slovenia	8.6	Croatia	7.0	Romania	5.5

Eurostat Statistics Explained [2]

An aging European population will undoubtedly result in an increase in the burden of chronic diseases in Europe, placing an enormous burden on national health budgets. The Global Action Plan for the prevention and control of non-communicable disease (NCDs) identified an overall goal for a 25% relative reduction in premature deaths (before the age 70) by 2025 [7]. This highlights the urgent need to identify cost-effective and evidence-based public health policies and interventions that are suitable for the European population, in order to help alleviate the burden of chronic diseases.

## The role of population cohorts and evidence based prevention

Europe has been a leader in developing large population cohorts that include collection of extensive biological samples. The two most prominent examples include UK Biobank that recruited 500,000 people aged 40–69 years between 2006 and 2010 from across the UK (http://www.ukbiobank.ac.uk), and the EPIC cohort that undertook recruitment of 521,000 participants from 1993–1999 in 10 European countries, with study participants mostly invited from the general population in an age range of 35–70 years (http://epic.iarc.fr/). As of 2007, in the framework of the European ESFRI programme of the EC, a large fraction of the European cohorts (mostly population biobanks), clinical biobanks and twin registries have established the European biobanking infrastructure BBMRI (Biobanking and Biomolecular Research Infrastructure), which has obtained a formal ERIC (European Research Infrastructure Consortium) status in 2013. Participants of the UK Biobank study and several other recent population biobanks, underwent detailed health and lifestyle interviews and provided blood, urine, saliva and more recently occasionally even stool samples for future analyses. Follow-up for disease outcomes including cancer, cardiovascular and respiratory diseases is by a number of mechanisms. A novel feature of UK Biobank and other contemporary biobanks is that their primary purpose is to be used as a scientific resource by ‘external’ investigators in the scientific community anywhere in the world [8]. The future promise of such resources for health research can be illustrated with the achievements of the EPIC cohort: in the last 25 years, over 80,000 newly incident cancer cases, 18,000 cases of ischemic heart disease (IHD), 6000 cerebrovascular accidents and 14,000 cases of Type 2 diabetes have been reported, producing over 600 scientific publications 400 of which making use of the stored biological samples.

**Fig. 2:**
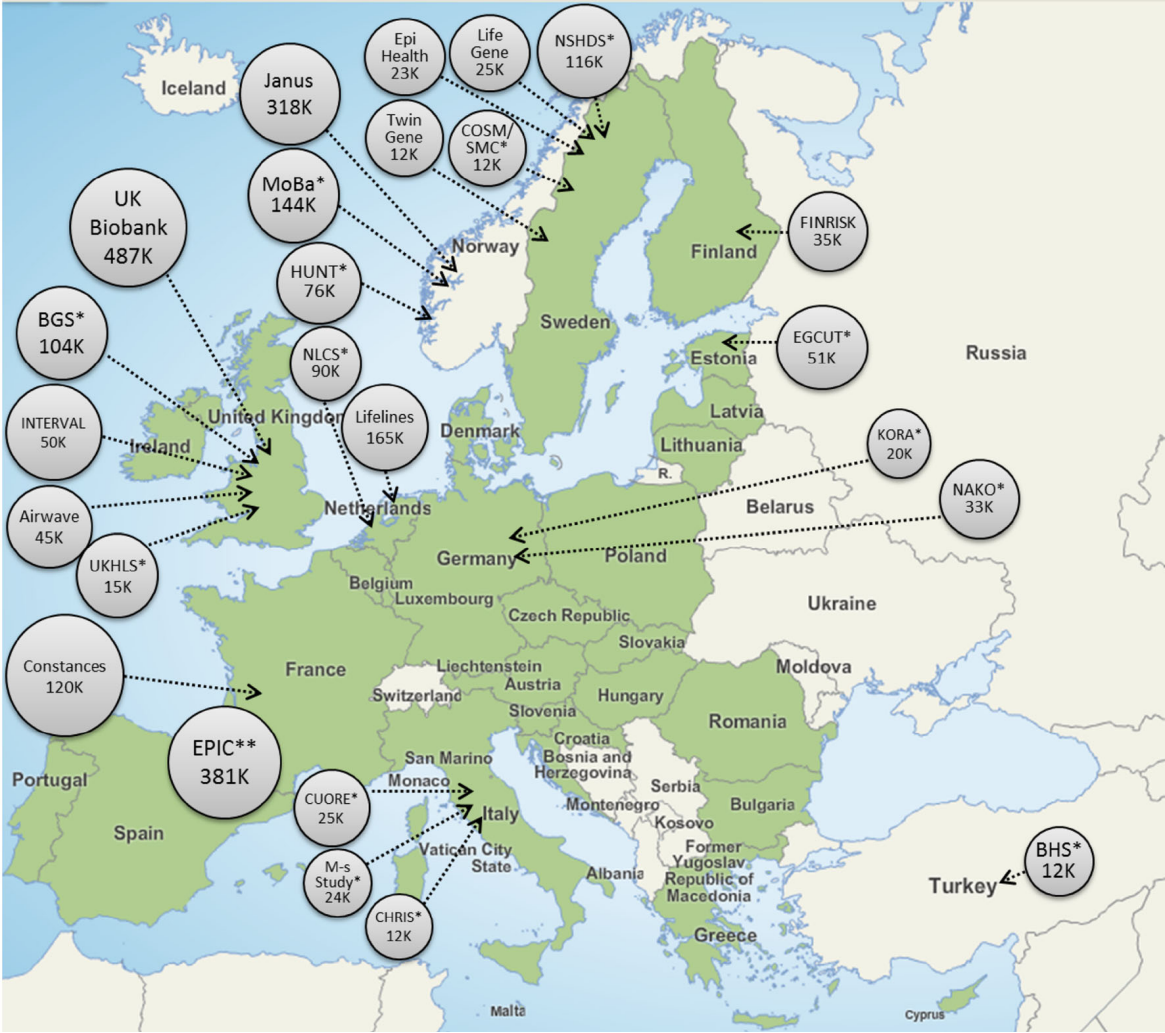
Prospective European cohorts with at least 10,000 participants and including baseline collection of biological samples. *Cohort acronyms by country: Estonia: EGCUT = Estonian Genome Centre, University of Tartu; Germany: KORA = KOoperative gesundheitsforschung in der Region Augsburg, NAKO = German national cohort; Italy: CUORE = Cohort of Italian Adult Women and Men, M-s study = Moli-sani Study, CHRIS = Cooperative Health Research in South Tyrol Study; Netherlands: NLCS = Netherlands Cohort Study; Norway: MoBa = Norwegian Mother and Child Cohort Study, HUNT = Nord-Trøndelag Health Study; Sweden: NSHDS = Northern Sweden Health and Disease Study, COSM/SMC = Cohort of Swedish men/Swedish Mammography Cohort; Turkey: BHS = Balcova Heart Study; United Kingdom: BGS = Breakthrough Generations, UKHLS = The UK Household Longitudinal Study. **EPIC (The European Prospective Investigation into Cancer and Nutrition) includes the following centres: EPIC-Denmark (56 K), EPIC-France (20 K), EPIC-Germany (Potsdam & Heidelberg) (50 K), EPIC-Greece (28 K), EPIC-Italy (47 K), EPIC-Netherlands (36 K), EPIC-Norway (9 K), EPIC-Spain (39 K), EPIC-Sweden (53 K), and EPIC-United Kingdom (43 K)

The more recent cohorts mostly have sample sizes between 10,000 and 100,000 individuals (Fig. 2), while in the last 10 years a new phase of cohort development emerged, with even > 100,000 participants. The total size of population cohorts in Europe stands at over 2.5 million participants, although with a strong disparity in cohort coverage, mainly due to economic restraints. Western and Northern Europe are strongly covered, including up to 5% of the entire population in some countries like Finland and the Netherlands, while in Eastern Europe large population cohorts are rare.

Much of our understanding of the driving forces of ill-health and premature mortality derive from analyses of these large cohorts. The second half of the 20th century was characterised by a substantial improvement in public health and life expectancy in practically all parts of the world [9]. When Jerry Morris wrote ‘Uses of epidemiology’ in 1955 [10], one-third of men aged 35 in the UK could be expected to die before the age of 65, whereas that figure is now less than 15%. The reasons for this are partly clinical, including better treatment and access to care, and partly societal, including better housing, vast reductions in road traffic accidents and improved nutrition, although a substantial part is due to the demonstration of the important negative effects of tobacco smoking on lung cancer and subsequently over 19 other cancers, as well as numerous other maladies including common vascular and respiratory diseases [11]. The weight of the evidence has been from population based cohort studies that minimise certain biases and allow for accurate estimation of absolute risk effects. The 2004 IARC monograph evaluation of tobacco smoke and involuntary smoking [12] included results from over 40 different cohorts including 13 from Europe, 19 from North America and 12 from Asia. Similarly, we now have accurate estimates of the role of other important risk factors on public health outcomes, including for elevated body mass and hypertension [13]. Although a potential beneficial effect of alcohol at low doses is still debated, the role of excess alcohol consumption in premature mortality has also been evaluated through large cohort studies, with a particular focus on populations in Russia and east Europe where large numbers of adults drink to excess [14]. Cohorts that focus on specific subgroups such as workers have been fundamental in identifying occupational exposures that lead to cancer and other outcomes, and have resulted in much improved workplaces [15]. Cohorts that focus on children have also been instrumental for identifying key stages of childhood that are instrumental for healthy development, as well as more specific outcomes such as the role of sleeping position in sudden infant death syndrome (SIDS) [16, 17]. Large population cohorts are therefore the basic tool of epidemiology and population health, and even though many of our health indicators have improved over recent decades they remain as essential as ever in evaluating the various ways in which our rapidly changing society can impact on health.

## Using cohorts to assess the population impact of known risk factors and identify novel risk factors

A recent example of how European cohorts can contribute with evidence relevant for policy making is an analysis from 265,000 individuals from the EPIC cohort with complete risk factor data, 11,930 of whom died during the follow-up before the age of 70. Six common risk factors could collectively explain 57% of premature deaths (Table 3) [18]. These data illustrated the predominant impact of tobacco smoking which explained 31% of premature deaths. Further, smokers with otherwise healthy lifestyle characteristics had similar death rates as did never smokers who presented multiple “unhealthy” risk factors, including a poor diet, obesity, hypertension, physical inactivity and consumption of more than two alcoholic drinks per day [18]. Overall, over 95% of non-smokers with additional healthy characteristics reached the age of 70, whereas only 80% of female smokers with additional unhealthy characteristics and 64% of men reached the age of 70. Another study, in Estonia and Finland, has highlighted that a large portion of short-term all-cause mortality risk can be caught in the combined levels of just 4 NMR-metabolic biomarkers [19]. These studies clearly demonstrate how cohort data can be used to evaluate the relative importance of common risk factors or readily measurable metabolites for the risk of premature death. This information would be highly relevant to public health measures to curb premature death in Europe.

**Table 3:** Population attributable fractions (AF) of pre-mature mortality in 10 European countries for common risk factors

Risk factor	AF (%)	95% CI
Tobacco smoking	31	(31–32%)
Poor diet	14	(12–16%)
High waist-to-hip ratio	10	(8–12%)
High blood pressure	9	(7–11%)
Physical inactivity and low physical activity	7	(5–9%)
High alcohol use	4	(3–4%)
Combined	57	(55–59%)

Muller et al. [18]

Large population cohorts that cover heterogeneous populations with a diverse range of exposures and chronic disease incidence are also the most appropriate setting for further elucidating the unknown causes of chronic disease. Even while diseases such as cancer have been extensively studied, many causes are still unknown, with only about 50% of cancer incidence accounted for by known causes [20]. While it is likely that a proportion of cancers, and perhaps even other chronic diseases, do have a true stochastic pathology, it is also clear from international disease trends and differences that important unknown causes remain [21]. A co-ordinated analysis of suspected causes across numerous population cohorts does however require that an important component of standardization is undertaken prior to any analysis being feasible.

## Behaviour modification and large scale studies

Exposures and lifestyle patterns are generally not static but change over time. A clear example is that of smoking, with many individuals who take up the habit, usually in the late-teens, quitting smoking in middle or late age. Past information on smoking habits is relatively easy to obtain from questionnaires when individuals are recruited into cohorts, resulting in accurate and risk estimates of past smoking habits and quitting at various ages [22]. This is not however the norm, and a limitation of many cohorts is that they are restricted by the information that is gained at study recruitment, with often minimal amounts of information regarding historical changes in many other types of exposure. Multiple interviews over time can help to identify changes in future exposure status, although they are not the norm for cohort studies, primarily because of cost and the difficulty in getting study participants to agree to multiple re-interviews. Cohort studies are therefore not ideal for measuring the effect of changing exposure over time.

Given the observational nature of cohort studies, other important limitations include confounding, confounding by indication and reverse causation. The impact of representativeness has also been discussed at length recently, and is of particular relevance given that some very large cohorts such as UK Biobank have participation rates of less than 10% of those invited to participate [23–25]. The potential for collider bias, whereby exposures and additional risk factors are associated with the probability of inclusion, may be underappreciated. An additional concern is that many findings related to protective effects of diet and nutritional components have not been replicated in subsequent randomized studies, indicating that the initial findings were due to confounding. Reverse causation is generally thought to be a limited problem for prospective studies, although recent genetic evidence from genes that correlate with alcohol consumption would seem to suggest that the much vaunted protective effect of moderate levels of alcohol on cardiovascular disease may be better explained by individuals prone to developing the disease avoiding alcohol altogether [26]. One alternative to overcome this challenge is through building large scale randomized studies into prospective cohorts. Although randomized studies that subsequently repurpose themselves as cohorts are relatively common, the opposite of conducting randomized trials within established cohorts is much rarer. One positive example is the Golestan cohort in Iran of 50,000 individuals that has a trial of cardiovascular disease mortality reduction using a polypill [27].

## Cohort studies in the era of precision medicine

The potential for population cohorts to contribute to the understanding of why some individuals develop specific diseases, and how they respond to particular treatments is the primary rationale behind the recent initiative in the US to build a national cohort of one million US citizens [28]. Central to the research into human biological variation which is underpinning the drive towards personalized medicine, is the genotyping of hundreds of thousands of genetic variants across the human genome in millions of people, a hitherto unprecedented scale. This allows for evaluation of the vast majority of genetic variation due to common single nucleotide polymorphisms as in genome-wide associations studies (GWAS). One important aspect of the US ‘Precision Medicine’ initiative was to provide genetic data on a large number of people with extensive phenotype information and clinical follow-up. Development of this large cohort of one million adults in the US is currently underway.

Genetic research into common diseases in Europe is on the verge of another period of rapid discovery due to the genotyping of large numbers of cohort participants. The most notable is the genotyping of all 500,000 participants in UK Biobank using a genome-wide genotyping array. As of July 2017, genome-wide data on all 500,000 individuals has been made available to the scientific community, with additional exome sequencing of at least 50,000 individuals underway. The UK Biobank is performing additional phenotyping for a large panel of circulating biomarkers. The advent of large-scale genome-wide data has opened up several avenues for innovative research aiming to understand causes and mechanisms underlying complex diseases. These include Mendelian randomization type studies that provide additional evidence on the causal relevance of lifestyle risk factors in the absence of the various biases that are sometimes difficult to exclude in traditional observational epidemiology [29].

Cohort studies have had their greatest success for exposure that are relatively stable and easy to measure (e.g. smoking and obesity), whereas ubiquitous environmental exposures or those occurring during specific time periods have been a lot more problematic to study. The developing fields of lifecourse epidemiology and exposomics, whereby the totality of environmental exposures from conception onwards are evaluated, is a novel and exciting approach to studying the role of the environment in disease development [21, 30].

## The importance of population cohorts for identifying individuals at high risk of disease

Another key aim of the precision medicine paradigm in the context of disease prevention is developing methods and tools that allow identifying individuals who are likely to develop specific diseases in the near future. For instance, being able to identify particular subgroups or even individuals who are at high risk of imminent cardiovascular events would allow intense interventions, with the potential of saving many lives across Europe. Evaluation of the feasibility, usefulness, safety and cost-effectiveness of CT screening is currently ongoing in the Swedish CArdioPulmonary BioImage Study [31]. Another aspect of individual disease prevention is improving the identification of individuals that are most likely to benefit from cancer screening. There are currently a number of screening programs in place in many European countries, in particular for breast cancer (by mammography), cervical cancer (by cervical pap-smear) and colorectal cancer (by faecal occult blood test followed by recto-sigmoidoscopy or colonoscopy), with lung cancer screening also being considered. Detecting these cancers through screening at pre-neoplastic or early stages when they remain curable can measurably reduce their mortality. On the other hand, the screening efficacy, including number of subjects needed to screen to detect a cancer and the subsequent cost implications has generally been less than optimal. There are also negative effects of screening, including the markedly high risk of false positives with subsequent clinical follow-up and possible iatrogenic consequences. For instance it has recently been demonstrated that it is possible to reduce lung cancer mortality by 20% through early detection [32]. However, the NLST study also highlighted several important negative aspects associated with CT screening in terms of morbidity associated with overdiagnosis, treatment of benign nodules and financial costs. Furthermore, many lung cancer cases do not satisfy the NLST screening criteria, and are therefore not eligible for screening. Within two large European prospective cohort studies (UK Biobank and EPIC) it has been estimated that only about 50% of the incident lung cancer patients were eligible for screening according to the NLST criteria [32]. Taken together, these data clearly illustrate the urgent need to improve the eligibility criteria for CT screening using comprehensive risk prediction models, and multiple studies suggest that incorporating biomarkers of lung cancer risk could substantially improve such models. Prospective cohorts will have a key role in providing prediction tools for common chronic diseases given the need for pre-diagnostic data and biospecimens. Candidates for blood based early detection markers including circulating tumour DNA [33], recently the focus of a large private initiative, micro RNA and protein based biomarkers [21]. Incorporation of technological expertise from biotech companies will likely be fundamental to translating scientific developments in the area into practical screening tools. While moving ahead with development of prediction tools for identifying high risk individuals, it will also be important to ensure that the limitations of this approach are not forgotten. As discussed over 30 years ago by Geoffrey Rose, there will remain a need to also undertake a broad population approach to prevention, where one attempts to modify the risk distribution among all [34].

## The importance of collaboration

Prospective cohort studies are instrumental in evaluating the impact of a wide range of risk factors for specific diseases, as well as for developing disease-prediction tools. However, because they recruit study participants prior to disease onset and follow them throughout the life-course, prospective studies need to be of very large size with adequate follow-up time to be able to study common diseases in a reliable manner. Moreover, studying all but the most common diseases is challenging in single prospective cohorts. Evaluating the consistency in study results across different populations will also be crucial in assessing the importance of promising findings, in particular when considering translating them into public health measures. To maximize achieving this, it is crucial to make European cohorts compliant with the FAIR and FAIR-Health principles, where FAIR stands for ‘Findable, Accessible, Interoperable and Reusable’ [35], and the ‘health’ extension refers to additional requirements for health data of human subjects: traceable quality and reproducibility records and compliance with national and international privacy protection principles. These latter have been developed within BBMRI-ERIC (Holub et al., manuscript submitted) anticipating on consistency with the upcoming European Open Science Cloud.

## Cohort infrastructure: needs and gaps

The UK Biobank study as well as several large international initiatives like ENGAGE [36], GIANT [37] and CHARGE [38] have led the way on developing or integrating large population cohorts with extensive lifestyle, genomic and metabolomics characterization, and making materials and data available to the broad scientific community. While the large national biobank are exemplary in their efforts, on their own they will not be sufficient for the broad European public health and research community. As demonstrated above, the UK is but one aspect of the European public health experience, and even a cohort of 500,000 will have limitations with respect to study size. Initiatives like EPIC have significant strengths in this regard, covering many countries in Europe and with sometimes very long follow-up and often extensive genomic and more recently metabolomic characterization. Other large new national cohorts are under recruitment, and will complement the established initiatives (Fig. 2). In order to initiate discussion regarding how population cohorts across Europe can work together, leaders of these cohorts have joined together to initiate an informal ‘European cohort consortium’ (ECC). This partnership includes 40 cohorts ranging in size from 10,000 up to 520,000 individuals and with a total potential sample size of over 2.5 million individuals.

If Europe is to retain a leadership position in population based health research, as well as continue to provide evidence for improving public health, then several key actions need to be undertaken. First of all, mechanisms need to be established that allow studies to be conducted across these cohorts. These include efforts to ensure access to outside investigators, as well as initiatives to harmonize lifestyle and exposure data, as well as outcome measures for morbidity and mortality. These cohorts need to be viewed as an essential European research resource, contributing to the research facilities under construction in the BBMRI-ERIC infrastructure, including coordinated ELSI (Ethical, Legal and Social Implications) and IT platforms, a common sample and metadata catalogue and standardized quality assessment and control. The true value of these cohorts will only come through extensive genetic and phenotypic characterization. Assays for broad panels of genetic, phenotypic and infectious markers are becoming cheaper, especially when done on a large series. Further, one needs to recognize that large parts of Europe do not have large established population cohorts, in particular in central and Eastern Europe, and efforts need to be made to fill this gap.

Finally, we appreciate that such a bold initiative will only go ahead if there is political will to support it. The management and development of large cohorts require considerable resources, with both EPIC and UK Biobank costing in the region of 100 million euros to establish. While modest national investments have been possible in the establishment of national BBMRI nodes and the national contributions to BBMRI-ERIC, none of this reflects the actual foundation cost of each nation’s biobanks proper, let alone the essential investments needed for maintenance and innovations. The US national cohort has been allocated $130 million for the current budget year alone. Europe is in the envious position where many of the cohorts are already in place, and what is required is a structure to bring them together. Given the scientific potential of such a large European cohort initiative, as well as the importance of informing public health policy across the continent, a more relevant question may be whether we can afford not to build it.
